# Clinical features of diabetes mellitus in hereditary pancreatitis

**DOI:** 10.1186/1758-5996-7-S1-A107

**Published:** 2015-11-11

**Authors:** Marcio Garrison Dytz, Pedro Arthur Hamamoto Marcelino, Ana Luiza Campanholo, Isabella Albuquerque Pinto Rebello, Flavia Lucia Conceição, Tânia Maria Ortiga-Carvalho, Lenita Zajdenverg, Melanie Rodacki

**Affiliations:** 1 Hospital Universitario Clementino Fraga Filho, Rio de Janeiro, Brazil

## Background

Hereditary pancreatitis (HP) is a rare autosomal dominant disease characterized by recurrent acute pancreatitis that progresses to chronic pancreatitis. Common clinical manifestations are abdominal pain, disabsorptive syndrome due to exocrine dysfunction and diabetes mellitus (DM) due to damage to islet cells.

## Objective

To describe the characteristics of DM secondary to HP in a family with mutation in PRSS1 gene.

## Materials and methods

Patients of one family with DM secondary to HP due to N29T mutation in exon 2 of the PRSS1 gene were evaluated by review of medical records and download of CG from Accucheck Active glucometer via software Accucheck 360º. Glycated hemoglobin (HbA1c) during the follow up and capillary blood glucose (CBG) in the previous month were analyzed.

## Results

**Figure 1:**
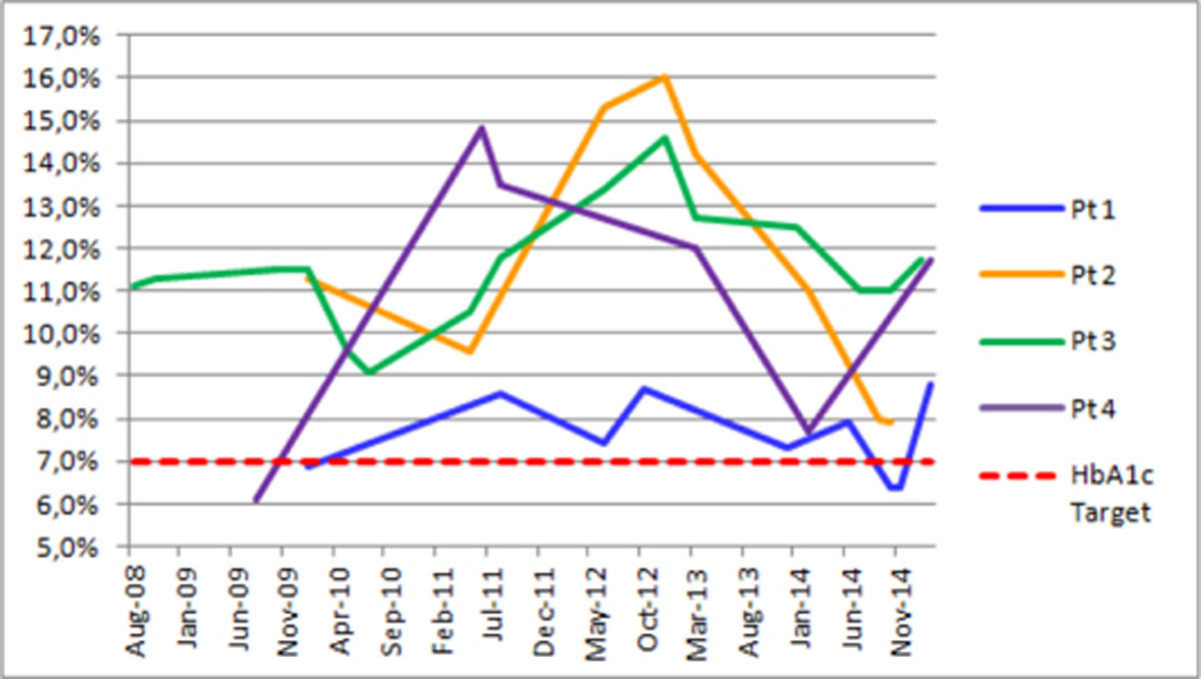
A retrospective follow-up indicated that HbA1c varied widely since DM onset and/or the last 6 yrs.

Five patients were included. In 3 patients, the diagnosis of HP preceded that of DM, while in 2 the opposite occurred. The average time between diagnosis of HP and DM was 80±24 months (range: 60-180 months). All patients used insulin. The mean dose was 0.71±0.63 IU/kg (range: 0.27-1.76 IU/kg). In four patients, we observed the use of other drugs (Metformin and glibenclamide) before insulin therapy was started, for a mean time of 46±45 months (range: 4-96 months). No patient presented HbA1c lower than 7%, one patient presented HbA1c between 7% and 9% and 4 patients presented HbA1c higher than 9%. The average home capillary blood glucose was 217.00±69.44, ranging between 145 and 306 mg/dL. The average standard deviation (SD) of capillary blood glucose (SD) was 104.75±15.56, ranging between 94 and 127 mg/dL. The average duration of diabetes was 120.80±80.32 months, ranging between 20 and 228 months. A retrospective follow-up indicated that HbA1c varied widely since DM onset and/or the last 6 yrs., as shown in figure 1. The SD of HbA1c over this period ranged from 1.0 to 3.2 (mean: 2.6%). In 2 patients, diabetic chronic complications (retinopathy and diabetic neuropathy) were observed.

## Conclusion

DM secondary to HP may appear before or after the exocrine manifestations of pancreatitis. In this family with mutation in PRSS1 gene, glycemic control was poor and labile, with important glycemic fluctuations. Insulin was necessary in all cases, with a wide dosage variation between the different family members. These data indicate that DM secondary to HP has difficult management. Strategies to improve the glycemic control in affected patients should be pursued.

